# Fucoxanthin and Polyunsaturated Fatty Acids Co-Extraction by a Green Process

**DOI:** 10.3390/molecules23040874

**Published:** 2018-04-11

**Authors:** Antoine Delbrut, Pierre Albina, Théo Lapierre, Rémi Pradelles, Eric Dubreucq

**Affiliations:** 1 Montpellier SupAgro, UMR 1208 IATE, 2 Place Viala, 34060 Montpellier CEDEX 2, France; antoine.delbrut@supagro.fr (A.D.); pierre.albina@outlook.fr (P.A.); theo.lapierre@etu.umontpellier.fr (T.L.); 2 Microphyt, 713 Route de Mudaison, 34630 Baillargues, France; remi.pradelles@microphyt.eu

**Keywords:** polyunsaturated fatty acids, fucoxanthin, *Tisochrysis lutea*, *Phaeodactylum tricornutum*, green solvents, food, cosmetics, microalgae

## Abstract

By their autotrophic nature and their molecular richness, microalgae are serious assets in the context of current environmental and societal challenges. Some species produce both omega-3 long chain polyunsaturated fatty acids (PUFAs) and xanthophylls, two molecular families widely studied for their bioactivities in the fields of nutrition and cosmetics. Whereas most studies separately deal with the two families, synergies could be exploited with extracts containing both PUFAs and xanthophylls. The purpose of our work was to determine cost effective and eco-friendly parameters for their co-extraction. The effect of several parameters (solvent, solvent/biomass ratio, temperature, duration) were studied, using two microalgal species, the non-calcifying Haptophyta *Tisochrysis lutea,* and the diatom *Phaeodactylum tricornutum*, that presents a silicified frustule. Analyses of PUFAs and fucoxanthin (Fx), the main xanthophyll, allowed to compare kinetics and extraction yields between experimental protocols. Co-extraction yields achieved using 96% ethanol as solvent were 100% for Fx and docosahexaenoic acid (DHA) in one hour from *T. lutea* biomass, and respectively 95% and 89% for Fx and eicosapentaenoic acid (EPA) in eight hours from *P. tricornutum*. These conditions are compatible with industrial applications.

## 1. Introduction

According to the World Health Organization (WHO), 2.8 million people die every year from obesity-related diseases, a number that tripled between 1975 and 2016. The WHO has recorded 1.9 billion overweight and 650 million obese people in 2016 [1]. Overweight and obesity have been estimated to account for about 65–80% of new cases of type 2 diabetes [2]. The prevalence of overweight and obesity has also increased globally in children and adolescents from 8.5% to 15%. In 2010, overweight and obesity were estimated to cause 3.4 million deaths, 3.9% of years of life lost, and 3.8% of disability adjusted life-years worldwide [3]. As part of a balanced diet and exercise routine, food supplements could provide an important support to persons looking to manage their weight [4,5,6,7]. In this context, specifically developed bioactive compounds could offer significant metabolic effects that can contribute to weight management and weight loss.

Among the very large diversity of compounds found in microalgae, two molecular families are of peculiar interest in the fields of nutrition and especially in the fight against overweight. The first consists of ω-3 long chain polyunsaturated fatty acids (PUFAs), of which eukaryotic algae, especially microalgae, are primary producers. PUFAs are considered beneficial against obesity [8,9]. First, their consumption is important for the ω3/ω6 balance, which is largely in favor of ω6-PUFAs in developed countries. This imbalance seems to be related to obesity, regardless of the number of calories ingested [10]. Indeed, while a 1:4 proportion is considered as optimal, its value is 1:15 in Western Europe and more than 1:16 in the United States [11]. In addition, numerous studies on several models have shown metabolic effects of PUFAs related to obesity, such as the reduction of markers of inflammation in plasma of obese women [8], the reduction of fat accumulation in mice fatty tissues [9] or the in vitro suppression of adipocyte differentiation [12].

The second molecular family of interest are carotenoids. These compounds are synthesized by all photosynthetic organisms and by some bacteria and fungi [13]. Carotenoids are divided in two major subfamilies, carotenes and xanthophylls. The latter are oxygenated derivatives of the former and are more diversified in algae [14]. Some xanthophylls, such as astaxanthin, cantaxanthin or fucoxanthin, have been only found in algae, bacteria and yeast. Fucoxanthin (Fx) has a unique structure, including an allenic bond, an epoxy and carbonyl groups, that confers many biological activities to this molecule [15,16]. Some of them are shared with PUFAs, such as antioxidant activity [17], effects on cognition [18], anti-inflammation functions on mice [19], or even anti-obesity activities on mice [20] and humans [7]. These activities may also at least partially be related to Fx metabolites. For example, Fx is converted into fucoxanthinol before being stored in liver and other tissues in humans [21] and also into amarouciaxanthin A in fat tissues from both mice and rats [22]. The anti-obesity activities of Fx have been particularly well described and studied on mouse and in vitro cells. Gammone and D’Orazio [22] have surveyed the main mechanisms of action described for Fx and/or its derivatives against obesity: reduction of plasmatic and hepatic triglyceride concentrations, reduction of fatty acid synthesis, decreased adipocyte differentiation, inhibition of glucose uptake in mature adipocytes, stimulation of thermogenesis and lipolysis in white adipose tissue, and the regulation of leptin, a hormone linked to the reduction of dietary intake and an increase in the use of lipid reserves.

As mentioned above, Fx and PUFAs share some common effects against obesity. For example, Fx [23] and PUFAs [12] inhibited the expression of same several transcription factors and genes (PPARγ, C/EBPα, and SREBP1c) linked to adipocyte differentiation on the same in vitro study model. These two molecular families within the same extract could therefore have synergetic actions expected to provide unequalled benefits on metabolic mechanisms involved in weight loss. To our knowledge, no published studies have tested the combined effects of Fx and PUFAs on anti-obesity activities, but the unique ability of some algae to co-produce Fx and ω3-PUFAs may be highly interesting. Microalgae have generally high production costs and very few are currently approved for human consumption. Nevertheless, they feature many advantages over macroalgae, especially when production is carried out in closed systems: carotenoid and PUFAs can reach levels 10 to 20 times higher and stable throughout the year, growing conditions are controlled to ensure a high productivity, and pollutants (heavy metals, pesticides) and biogenic contaminants can be controlled [14,24].

The extraction of both lipids and xanthophylls from a same biomass has been reported for macroalgae [16,25,26,27] and microalgae [28,29]. The extraction solvents used were chloroform, methanol, dimethyl ester and/or hexane, or, in one case, supercritical CO_2_. Indeed, at lab scale, total lipids are traditionally extracted with methanol and chloroform according to Folch et al. [30] or Bligh and Dyer [31]. On a larger scale, lipid extractions are more commonly performed using hexane [32] or supercritical fluids [33]. However, hexane is not a good solvent for Fx extraction [34,35,36]. Sivagnanam et al. [16] have shown that supercritical CO_2_ extraction is the best compromise for lipid and Fx extraction compared to acetone, hexane and ethanol. Nevertheless, this method requires expensive investments. The solvents known to efficiently extract Fx from microalgae are ethanol, methanol, acetone and ethyl acetate [34,35,36]. These four solvents are, among a list of the 26 most commonly used in the pharmaceutical industry, the ones with the lowest negative impacts according to a study by Capello et al. [37] that defined green solvents as those that minimise energy demand and their negative impact on the environment, safety and health. These parameters are now commonly used to calculate scores in order to classify green solvents [38]. It is to be noted that in spite of its favorable classification as a green solvent, methanol is not authorized for use in nutrition and cosmetics due to its toxicity.

In the present work, the kinetics of Fx and total PUFAs co-extraction were compared in the presence of several green solvents (96% and 80% ethanol and 100% ethyl acetate) and different solvent/biomass ratios at various temperatures, with the aim of achieving a process safe for users and the environment, and compatible technically and economically with nutrition and cosmetic markets. To our knowledge, this is the first time that the co-extraction conditions of these two molecular families have been optimized, moreover by an eco-friendly and cost effective process. Another constraint on the solvent was that it should be able to solubilize compounds characterized by a large range of logP values (logarithm of the octanol/water partition coefficient). In the absence of consistent literature data, COSMO-SAC (COnductor like Screening MOdel—Segment Activity Coefficient) calculations [39,40] were performed for fucoxanthin and model compounds from different lipid classes containing PUFA (free fatty acids, phospholipids, triacylglycerols) as an information on the polarity of the compounds of interest. The calculated logP values were 8.0 for docosahexaenoic acid (DHA), 10.0 for fucoxanthin, 13.5 for 1-oleoyl 2-docosahexaenoyl phosphatidyl choline and 24.2 for 1-oleoyl 2-docosahexaenoyl 3-palmitoleoyl glycerol.

The study was performed on microalgae grown at industrial scale in 5000 L tubular photobioreactors (PBR). These PBRs, described by Muller-Feuga et al. [41], ensure a co-circulation of the liquid medium and CO_2_-enriched air in order to optimize gas exchanges. In addition, they are designed to minimize mechanical stress, which guarantees the preservation of the most fragile microalgal species. They are placed in a greenhouse to regulate natural light and temperature. The two microalgae species chosen were *Tisochrysis lutea* and *Phaeodactylum tricornutum*. Both co-produce Fx and ω3-PUFAs. They were selected from 17 species based on bibliographic and experimental data (four were cultivated in our laboratory). The selection criteria were their productivity in Fx and PUFAs in the growing conditions tested. *T. lutea* is a Haptophyta almost free of cell wall [42,43] while *P. tricornutum* is a diatom with a silica frustule [44]. Although neither of the two species is currently authorised for human consumption, they were considered as good models to validate Fx and PUFAs co-extraction parameters.

## 2. Results and Discussion

### 2.1. Fx and ω3 PUFAs Contents

*T. lutea* and *P. tricornutum* were cultivated in 5000 L Camargue tubular photobioreactors at Microphyt production plant (Baillargues, France). Cultures were grown in an artificial marine environment in semi-continuous mode as described by Muller-Feuga et al. [41]. The biomass compositions presented in Table 1 are averages of three independent analyses on one batch of one kilogram (*T. lutea*) and 1.6 kg (*P. tricornutum*) of dry biomass. Linolenic acid (ALA), stearidonic acid (SDA), eicosapentaenoic acid (EPA), DHA and Fx contents were used as references (100% extraction) in the development of extraction procedures. For this section, PUFAs were determined by direct transmethylation on the dry biomass of both species. The solvents selected for the analytical determination of Fx from the two species were different (probably due to the very different nature of these species). Indeed, according to the literature [34] and in-house preliminary tests, 96% ethanol was selected as the reference solvent for analytical dosage of Fx in *T. lutea* rather than 100% methanol. In *P. tricornutum*, however, methanol was selected as the reference solvent for analytical dosage of Fx extraction rather than ethanol as also shown by Xia et al. [36].

Representative chromatograms, retention times and void times of the chromatographic system for Fx and PUFAs of both species are given in Figures S1 and S2 of supplementary data.

*T. lutea* is rich in ALA, SDA, DHA (87 mg·g^−1^) and Fx (19 mg·g^−1^), with SDA as the major PUFA. In contrast, this microalgae does not accumulate EPA, a result in accordance with literature [45,46]. Fx contents given in literature are very variable, ranging from one percent [47] to more than two percent [34] of biomass dry weight (DW). For this study, microalgae were cultivated at large scale in standard conditions, non-optimized for carotenoid or PUFAs accumulation. It is therefore logical that their contents corresponded to average literature values. *P. tricornutum* had an opposite PUFAs profile, with EPA as the only PUFA of significant importance (29 mg·g^−1^), also in agreement with literature [48,49]. Its Fx content (13.3 mg·g^−1^) was consistent with classical ranges (5 [47] to 16 [35] mg·g^−1^). Total PUFAs and Fx contents were respectively 2.3 and 1.4 times higher in *T. lutea* than in *P. tricornutum* (Table 1).

### 2.2. Solvent Selection

The solvents used in food and cosmetic applications must be safe for consumers, operators and the environment. Three eco-friendly solvents (96% and 80% ethanol and 100% ethyl acetate) have been tested on freeze-dried *T. lutea* biomass and two on *P. tricornutum*. DHA and EPA were used as PUFAs markers for the optimization of the co-extraction from *T. lutea* and *P. tricornutum* biomasses, respectively. Fx was used as a marker for xanthophylls because it was the main pigment in the two species [50]. Based on bibliography, ethanol was selected due to its high efficiency for Fx extraction from both *T. lutea* [34] and *P. tricornutum* [35,51]. Shang et al. [52] observed that 90% ethanol allowed a slightly better Fx extraction than absolute ethanol. For this study we therefore chose to test aqueous ethanol (80% and 96%, *v*/*v*). Besides its lower price than absolute ethanol, the presence of water in ethanol favors the extraction of Fx, a relatively polar xanthophyll due to its epoxy, carbonyl and hydroxyl functional groups. Ethyl acetate is less often cited than ethanol, but Kim et al. (a) [34] found it slightly more effective than absolute ethanol in extracting Fx. It was thus also selected for extraction tests. Conversely, Xia et al. [36] found that methanol was the most effective solvent for extracting Fx from another diatom, *Odontella aurita*, a result that was confirmed by us with *P. tricornutum* in a preliminary study. Methanol was therefore used as a reference in the study of the Fx extraction kinetics from this species. Acetone was not selected for our study mainly because of its physical properties (flash point of −9 °C, boiling temperature of 56 °C), which were identified as less appropriate in the industrial context than ethanol (13 °C and 79 °C) and ethyl acetate (−4 °C and 77 °C).

The effect of three extraction parameters was studied: the solvent used, the extraction duration and the microalga species. The constant parameters were the biomass preparation procedure (freeze-dried biomass grinded by mortar and pestle), and extraction conditions, using a 20:1 (*v*/*w*) solvent/biomass ratio at atmospheric pressure, 30 °C, in the dark, under a nitrogen atmosphere and with the addition of BHT.

#### 2.2.1. Fx Extraction

**Figure 1:**
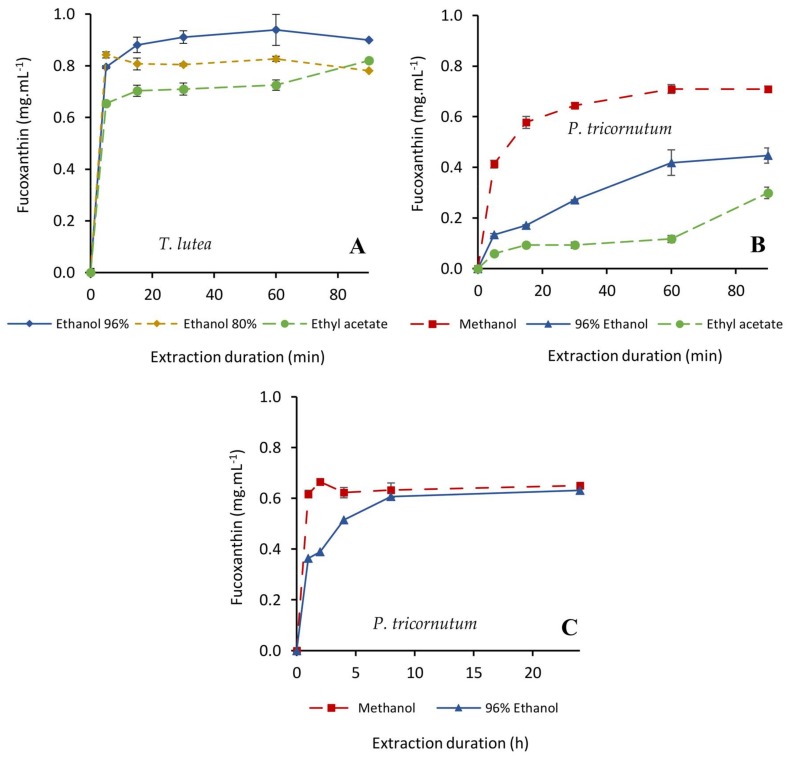
Kinetics of fucoxanthin extraction from freeze-dried *Tisochrysis lutea* and *Phaeodactylum tricornutum* with ethanol (80 or 96%), methanol (100%) and ethyl acetate (100%). Extractions were carried out for 90 min at 30 °C (**A**,**B**) and for 24 h at 40 °C (**C**) with a 20:1 solvent/biomass ratio (*v*/*w*) in a closed system with magnetic stirring (*n* = 3 replicates).

Fx extraction was very fast from *T. lutea* biomass, although with different kinetics according to the solvent used (Figure 1A). The highest Fx concentration (0.94 mg·mL^−1^) was achieved in 60 min with 96% ethanol, yielding better results than with 80% ethanol or ethyl acetate. Fx was much more difficult to extract from the diatom *P. tricornutum* (Figure 1B), with which 96% ethanol and ethyl acetate extracted only 0.45 and 0.30 mg·mL^−1^ of Fx, respectively, within 60 min. A same volume of methanol extracted 0.71 mg of Fx·mL^−1^, reaching a plateau after 60 min. Kim et al. [35] also found a better Fx extraction from *P. tricornutum* with ethanol than with ethyl acetate. Xia et al. [36] did not observe significant differences between methanol and ethanol for the extraction of Fx from the other diatom, *O. aurita*. However, in the case of *P. tricornutum* our comparative experiments have shown a different behavior, with 100% methanol as a better extraction solvent than 96% ethanol. For economic reasons and because Fx is a heat-sensitive molecule [53], the first tests were carried out at 30 °C for both species. However, increasing the temperature up to 40 °C to enhance Fx extraction [51] and increasing the extraction duration up to eight hours allowed to reach similar extraction yields from *P. tricornutum* biomass with 96% ethanol as compared to 100% methanol (Figure 1C).

**Figure 2:**
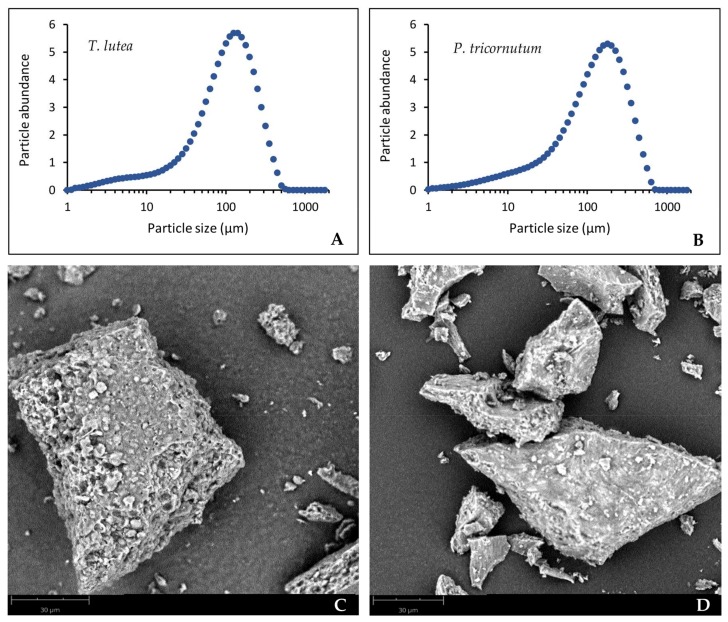
Determination of particle size distribution (graphs) and scanning electron microscope observations at 30 µm scale of freeze-dried biomasses of *Tisochrysis lutea* (**A**,**C**) and *Phaeodactylum tricornutum* (**B**,**D**) crushed with mortar and pestle.

The differences observed between ethanol and methanol extraction behaviors suggest a kinetic limitation related to solvent access to Fx within the biomass. The major difference in structure and composition between the two species is related to silica. Indeed, *T. lutea* is a so-called “naked” species because its cell wall is almost non-existent [42,43]. Conversely, the silica frustule *of P. tricornutum* [44] could modify favorably the partition coefficient of Fx between biomass and ethanol. Another possible effect of silica could be to cause a vitrification phenomenon during drying. To verify this, freeze-dried biomass samples prepared in the same conditions as for the extraction kinetics study have been observed with an electronic scanning microscope. As illustrated on Figure 2, the *T. lutea* dried samples displayed a slightly higher particle size (graph A) than *P. tricornutum* dried biomass (graph B). Moreover, *T. lutea* samples also appeared more porous (images C and D).

Although further investigations would be needed, this supports the hypothesis that differences in cell structure are probably a main contributor to the differences in extraction kinetics observed with the two alcohols.

#### 2.2.2. Extraction of PUFAs

**Figure 3:**
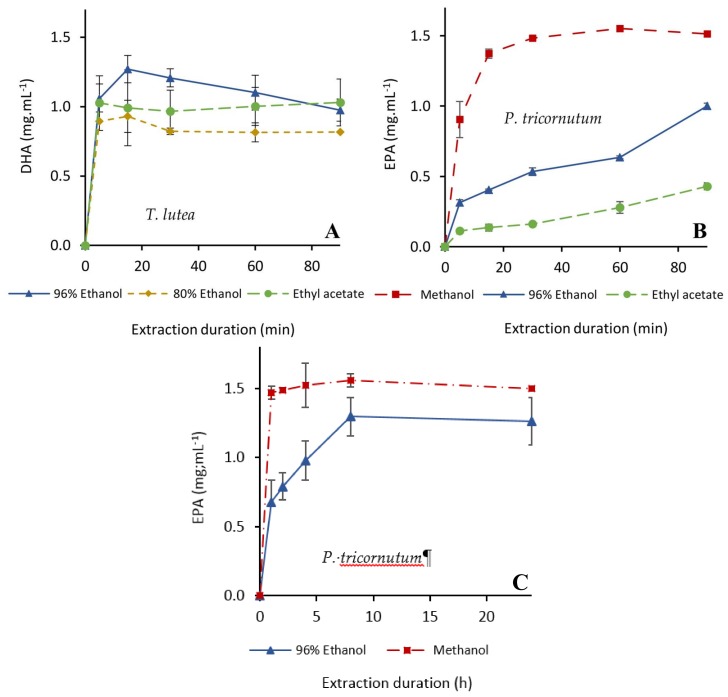
Kinetics of extraction of docosahexaenoic acid (DHA) from *T. lutea* (**A**) and eicosapentaenoic acid (EPA) from *P. tricornutum* (**B**) with ethanol (80 or 96%), methanol (100%) and ethyl acetate (100%). Extractions were carried out from freeze dried biomass for 90 min at 30 °C (**A**,**B**) and for 24 h at 40 °C (**C**) with a 20:1 (*v*/*w*) solvent/biomass ratio in a closed system with magnetic stirring (*n* = 3 replicates).

The kinetics of EPA and DHA extraction were very similar to those of Fx (Figure 3). DHA extraction from *T. lutea* biomass was very fast with 96% ethanol. Indeed, its concentration reached a maximum after 15 min (1.3 mg·mL^−1^) and then decreased over time (Figure 3A), maybe due to an exclusion from the solvent by other extractibles.

Using ethyl acetate resulted in a lower extraction yield (1.0 mg·mL^−1^) but the DHA concentration was stable over time. Results with 80% ethanol were logically unfavorable to lipid extraction because of the too high proportion of water. Extraction kinetics from *P. tricornutum* were similar for EPA and Fx. The maximum content (1.6 mg·mL^−1^) was achieved with 100% methanol in 60 min, while the equilibrium was still not reached after 90 min with 96% ethanol and 100% ethyl acetate (Figure 3B). A longer extraction at 40 °C with 96% ethanol did not allow to reach the extraction yields obtained with 100% methanol (Figure 3C), but a concentration of 1.30 mg EPA·mL^−1^ (89% extraction yield) in 96% ethanol was reached after eight hours at 40 °C.

The solvents tested thus showed very different efficiencies in terms of Fx, EPA and DHA co-extraction. The results strongly depended on the cell structure of the species, which modulates solvent accessibility to intracellular compounds. The best eco-friendly solvent tested was 96% ethanol, which was very efficient for both Fx and PUFAs extractions from *T. lutea* biomass. This solvent allowed to achieve the maximum extraction yield determined by the analytical reference method in less than one hour when using a 20:1 solvent/biomass ratio (Table 2). Freeze-dried *P. tricornutum* required long extraction durations and gave lower yields, especially for EPA (extraction yield of 89% in eight hours in the same conditions). The extraction yields were calculated by relating the concentrations of molecules in the different extraction conditions to the concentrations determined by the reference analytical methods (direct transmethylation on biomass for PUFAs, one hour extraction with 96% ethanol or 100% methanol to the 20:1 solvent/biomass ratio for Fx).

### 2.3. Ethanol/Biomass Ratio

Limiting solvent volume is a key optimization criterion that may represent environmental, security and economic benefits. For Fx extraction with ethanol, literature data suggest an optimal ratio of 20:1 (*v*/*w*) [36,51]. Lower ratios were thus tested to save ethanol volumes.

**Figure 4:**
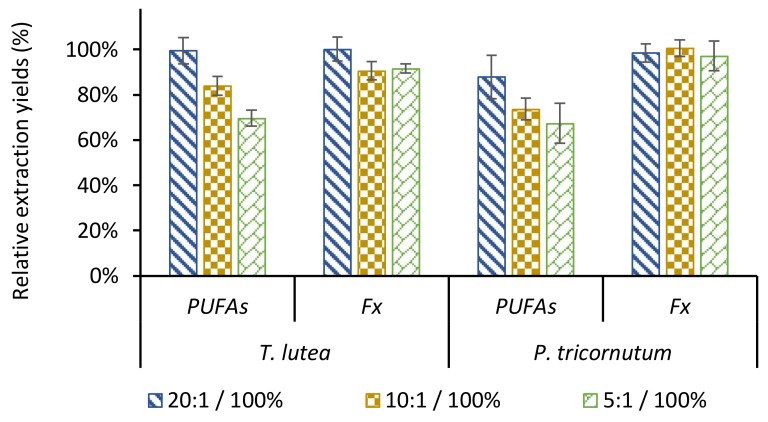
Comparison of the extraction yields of fucoxanthin (Fx) and total long chain polyunsaturated fatty acids (PUFAs) obtained with different ethanol/biomass ratios for 90 min at 30 °C (*T. lutea*) or for 24 h at 40 °C (*P. tricornutum*) with 96% ethanol in closed system with magnetic stirring. 100% references were determined from extractions with 96% ethanol (*T. lutea*) or 100% methanol (*P. tricornutum*) for Fx, and from direct transesterification of lipids from dry biomass into fatty acids methyl esters for PUFAs. Significant differences between data are represented by different letters. The relative ratios were compared for a given molecular family and species. (*p*-value ≤ 0.05, *n* = 3 replicates).

The parameters studied were the amount of biomass and extraction duration for both species. All other parameters remained unchanged. As shown in Table 2, lowering the ratio down to 5:1 (*v*/*w*) had similar effects on Fx extraction kinetics for both species, sustaining a good extraction yield (>90% of the maximum achieved with the 20:1 ratio) but requiring a longer extraction duration (up to 24 h with *P. tricornutum*) to reach the maximum. It is therefore possible to decrease the ethanol quantity without saturating the solvent for *P. tricornutum*. The maximum extraction yields for the three ethanol/biomass ratios tested on this species were not significantly different (Figure 4), thus validating the possibility of reducing the volume of ethanol by four for Fx extraction. The kinetics of PUFAs extraction were more impacted than those of Fx by the ethanol/biomass ratio. Data are summarized for EPA (*P. tricornutum*) and DHA *(T. lutea*) in Table 2, while extraction kinetics for all major PUFAs are given on Figure S3 of supplementary data. The maximum yields were achieved in 15 min with *T. lutea* biomass using a 20:1 ratio and in 60 min for the lower ratios. The same trend was observed with *P. tricornutum*. Figure 4 shows that the extraction yields depended significantly and inversely on ethanol/biomass ratios. This could be explained by the saturation of the solvent with extractibles. For an ethanol/biomass ratio of 10:1, the dry matter concentration in the ethanol extract from 100 g of biomass was 32.7 g/L with *T. lutea* and 26.7 g/L with *P. tricornutum*.

**Figure 5:**
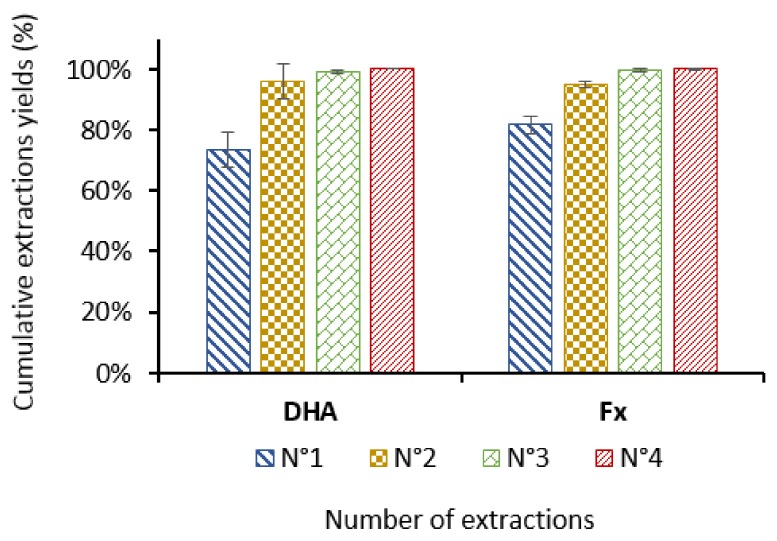
Cumulative extraction yields of fucoxanthin (Fx) and docosahexaenoic acid (DHA) from freeze-dried *Tisochrysis lutea* biomass with four successive 1 h extractions on the same biomass with a 10:1 (*v*/*w*) ethanol/biomass ratio (*n* = 3 replicates).

To improve the global yield, sequential extractions were performed using a 10:1 ethanol/biomass ratio on *T. lutea* biomass. At the end of each extraction, the biomass was separated from the solvent by centrifugation and extracted again with fresh 96% ethanol. As shown on Figure 5, two one-hour extractions resulted in the recovery of more than 95% of both Fx and DHA with *T. lutea* biomass.

These results suggest an optimal ratio around 10:1 for industrial-scale extraction because it allows (i) to reduce the quantity of solvent by two, thus limiting the volume to be handled and the size of the extraction tank, and (ii) to limit PUFAs and Fx losses as compared to the 5:1 ratio. With solvent recycling, the co-extraction of PUFAs and Fx from dry microalgal biomass thus appears as an economically viable method for industrial scale production.

## 3. Materials and Methods

### 3.1. Biological Material and Culture Conditions

The marine Haptophyta *Tisochrysis lutea* (CCAP 927/14) was provided by the Culture Collection of Algae and Protozoa (Oban, Scotland). The marine Bacillariophyta *Phaeodactylum tricornutum* (Mi136.M1.a) was isolated by Microphyt (Baillargues, France). The microalgae were grown in 5000 L photobioreactors (PBR) consisting in 1.2 km of glass tubes, each with co-circulation of liquid medium and CO_2_ enriched air [41,54]. PBRs were under a greenhouse allowing to control temperature (between 20 and 30 degrees all the year) and the intensity of natural light with curtains. The pH set points (7.2 for *T. lutea* and 8.2 for *P. tricornutum*) were automatically controlled by CO_2_ injection monitored by an inline pH probe (Fermprobe F-235, Broadley James, Silsoe, United Kingdom). Cultivations were conducted in a semi-continuous condition enabling to maintain the exponential growth phase during 67 days for *T. lutea* and 285 days for *P. tricornutum*. Cells were harvested by bowl centrifugation at 6000 rpm (KG 8006, GEA, Oelde, Germany) and concentrated at 18.9% (*P. tricornutum*) and 23.5% (*T. lutea*) of dry matter, frozen at −20 °C in plastic bags, freeze-dried and stored at −20 °C under nitrogen atmosphere. For this study, the dry biomasses contain residual salts.

### 3.2. Standards and Reagents

Fucoxanthin and glyceryl triheptadecanoate (TAG-17, used as an internal standard for each PUFAs assay) standards were from Sigma-Aldrich (Saint-Quentin-Fallavier, France), PUFAs from Larodan (Solna, Sweden) and extraction solvents (ethanol, methanol, ethyl acetate) from Carlo Erba (Peypin, France). Ultrapure water was produced using a Neptune Ultimate apparatus (Suez Water, Thame, UK).

### 3.3. Extractions

Freeze-dried biomass of the two species was crushed with mortar and pestle and extracted by 20 mL of solvent (96% ethanol, 80% ethanol, ethyl acetate for *T. lutea* and 96% ethanol, methanol, ethyl acetate for *P. tricornutum*) in a 50 mL glass bottles. Co-extraction of Fx and PUFAs was performed in an oven at 30 °C (*T. lutea* and *P. tricornutum*) or 40 °C (*P. tricornutum*) with magnetic bars agitation at 500 rpm for 5, 15, 30, 60 and 90 min (*T. lutea* and *P. tricornutum*) and 1, 2, 4, 8 and 24 h (*P. tricornutum*). Solvent/biomass ratios were 20:1, 10:1 or 5:1 (*v*/*w*) at constant volume of solvent. The extractions have been carried out in triplicate in darkness with nitrogen atmosphere and 0.1 mg·mL^−1^ BHT in order to limit oxidation.

### 3.4. Analyses

#### 3.4.1. Fucoxanthin

For *T. lutea* species, Fx was extracted with 96% (*v*/*v*) ethanol at 30 °C for 1 h, with a 20:1 solvent/biomass ratio (*v*/*w*). The maximum Fx extraction from *P. tricornutum* biomass was obtained using 100% methanol at 30 °C for 1 h with a 20:1 solvent/biomass (*v*/*w*). All the samples for Fx analysis were filtered on 0.20 µm Minisart filters (Sartorius, 25 mm) before injection in HPLC. Fx was analyzed by HPLC using a Waters Alliance 2695 system (Waters, Milford, MA, USA) equipped with a YMC (Kyoto, Japan) C30 column (100 A, 5 µm, 250 × 4.6 mm) thermostated at 30 °C. The mobile phase was 100% methanol (HPLC PLUS Gradient, Carlo Erba) at 1 mL·min^−1^. The injection volume was 10 µL and the runtime 15 min. Fx was detected at 450 nm by a UV/Vis Waters 2487 detector. Chromatograms were analyzed using the Masslynx 4.1 software (Waters). Calibration was performed with a Fx standard (Sigma-Aldrich).

#### 3.4.2. PUFAs

PUFAs were transmethylated by either a direct reaction with biomass, or after extraction and evaporation of the solvent under a nitrogen flux. Transmethylation was catalyzed by 5% (*v*/*v*) sulphuric acid in methanol for 90 min at 90 °C. Fatty acid methyl esters (FAMEs) were recovered in methanol/chloroform 1:2 (*v*/*v*) and injected in GC-FID for quantification. Analyses were performed using a Thermo Scientific Trace GC Ultra equipped with a Supelcowax 10 column (30 m × 0.320 mm, 0.25 µm of polyethylene glycol). The carrier gas was helium at 1.2 mL/min. The temperature program was: 150 to 250 °C in 10 min, then 11 min at 250 °C. Injector and detector temperature was 260 °C. FAMEs were detected by a flame ionization detector and quantified using the Chromcard software. Triheptadecanoylglycerol was used as internal standard. Injections of 1 µL were performed in split mode with a split ratio of 1:100. Compounds of interest were identified by comparing retention times with standards prepared in the same conditions.

#### 3.4.3. Particle Size Distribution

The granulometric distribution of biomass powders was analyzed using a Mastersizer 2000 laser granulometer (Malvern, Orsay, France) in a dry process in a Scirocco 2000 analysis cell. Particle sizes were evaluated by diffraction using two laser beams (red and blue).

### 3.5. Scanning Electron Microscopy (SEM)

Biomass powder samples were placed on a charge reduction hanger. Images were acquired using a benchtop Phenom Pro X scanning election microscope (Phenom World, Eindhoven, The Netherlands) with an acceleration voltage of 10 kV in image mode and a backscattered electron detector. 

### 3.6. Statistical Analysis

All data were obtained from three independent experiments. Statistical analyses were carried out using Excel 2016 software (Microsoft, Redmond, WA, USA).

### 3.7. COSMO-SAC Calculations

Molecular structures were drawn using MarvinSketch 2017 v.17.29.0 (Chemaxon, http://www.chemaxon.com). Using the ADF Modelling Suite 2017 v2017.12 (SCM, Theoretical Chemistry, Vrije Universiteit, Amsterdam, The Netherlands, http://www.scm.com), a first geometry optimization was performed by Density-Functional-based Tight-Binding using the DFTB3-D3-BJ model. The geometry was then refined and the distribution of the screening charges on the molecular surface calculated at the level of theory and with the parameter set consistent with the parametrization of COSMO calculations in ADF (Becke–Perdew functional, small core TZP basis set, relativistic scalar ZORA method, and good numerical integration quality) [39]. LogP were then calculated using the COSMO-SAC method with the parameters optimized by Chen et al. [40].

## 4. Conclusions

This study has shown that Fx and PUFAs can be co-extracted through an eco-friendly, cost effective and industrial-scale compatible process from microalgae. From *T. lutea*, 100% of Fx and DHA could be extracted in one hour, at 30 °C, using 96% ethanol with a solvent/biomass ratio of 20:1 (*v*/*w*). Two sequential extractions using a 10:1 (*v*/*w*) ratio (95% and 96% extraction efficiency for Fx and DHA respectively) can also be performed to limit the use of large volumes of solvent per batch. In *P. tricornutum* on the other hand, the nature of the cell wall caused a delay in the extraction kinetics and a loss of 22% of EPA with a solvent/biomass ratio of 20:1 (*v*/*w*) despite a higher extraction temperature (40 °C) and a longer extraction duration (24 h). For this species, an improvement prospect could be a dry biomass grinding to facilitate solvent access to the biomolecules of interest. Work is under way to test the nutritional and cosmetic combined activities of PUFAs and Fx.

## Figures and Tables

**Table 1:** Fucoxanthin (Fx) and long chain polyunsaturated fatty acids (PUFAs) contents ^1^ (mg·g^−1^ DW) in *Tisochrysis lutea* and *Phaeodactylum tricornutum* from a batch of biomass harvested at the industrial scale, as means of three independent extractions and analyses for each strain.

Compounds	*T. lutea*	*P. tricornutum*
Linoleic acid	17.8 ± 0.1	0.4 ± 0.1
Stearidonic acid	46.2 ± 1.4	0.4 ± 0.1
Eicosapentaenoic acid	1.8 ± 0.1	29.1 ± 1.3
Docosahexaenoic acid	23.0 ± 0.4	1.2 ± 0.1
Total PUFAs ω3	88.8 ± 1.8	31.1 ± 1.5
Fx	18.8 ± 0.5	13.3 ± 0.3

^1^ PUFAs were analyzed by direct transmethylation on dry biomass. The solvents used for Fx extraction was 96% ethanol for *T. lutea* and 100% methanol for *P. tricornutum*.

**Table 2:** Influence of solvent nature and the solvent/biomass ratio on the kinetics and yield of fucoxanthin (Fx) and long chain polyunsaturated fatty acids (PUFAs) extraction from freeze-dried *T. lutea* and *P. tricornutum* biomasses. Kinetics were followed during 90 min for *T. lutea* and 1440 min for *P. tricornutum*. Hyphens indicate that the target yield was not achieved. Where two values are indicated, the yield was reached between these two durations.

Species	Molecules	Extractions Parameters		Durations to Achieve Different Target Extraction Yields (min) ^1^		
		Solvent	Ratio Solvent/Biomass (*v*/*w*)	70%	90%	Maximum ^2^
*T. lutea*	Fx	96% ethanol	20:1	<5	15–30	60 (100%)
			10:1	<5	60	60 (90%)
			5:1	5–15	60–90	90 (91%)
		80% ethanol	20:1	<5	<5	5 (90%)
		100% ethyl acetate	20:1	5–15	-	90 (87%)
	Docosahexaenoic acid	96% ethanol	20:1	<5	5–15	15 (100%)
			10:1	15–30	-	60 (83%)
			5:1	30–60	-	60 (72%)
		80% ethanol	20:1	5	-	15 (73%)
		100% ethyl acetate	20:1	<5	-	5 (81%)
*P. tricornutum*	Fx	100% methanol	20:1	5–15	15–30	60 (100%)
		96% ethanol	20:1	120–240	240–480	1440 (95%)
			10:1	240–480	480–1440	1440 (97%)
			5:1	240–480	480–1440	1440 (91%)
	Eicosapentaenoic acid	100% methanol	20:1	5–15	15–30	60 (100%)
		96% ethanol	20:1	240–480	-	480 (89%)
			10:1	480–1440	-	1440 (77%)
			5:1	-	-	1440 (68%)

^1^ The extraction yields were calculated based on references methods (using 96% ethanol for Fx from *T. lutea*, 100% methanol for Fx from *P. tricornutum* and direct transmethylation on dry biomass for PUFAs). ^2^ Maximum yields and corresponding duration.

## Supplementary Materials

The following are available online. Figure S1: Chromatograms corresponding to ethanolic extracts of *P. tricornutum* and *T. lutea*, Figure S2: Chromatograms corresponding to direct transmethylation from biomass of of *P. tricornutum* and *T. lutea,* Figure S3: Influence of the solvent/biomass ratio (v/w) on the extraction kinetics and yield of fucoxanthin and long chain PUFAs.

## Abbreviations

ALA	Linolenic acid
BHT	Butylated hydroxytoluene
DHA	Docosahexaenoic acid
DW	Dry weight
EPA	Eicosapentaenoic acid
FAMEs	Fatty acid methyl esters
Fx	Fucoxanthine
GC-FID	Gas chromatography with flame ionization detector
HPLC	High performance liquid chromatography
logP	octanol/water partition coefficient
PUFAs	Long chain polyunsaturated fatty acids
SDA	Stearidonic acid
SEM	Scanning electron microscopy.
