# Supplementary material for: Fucoxanthin and Polyunsaturated Fatty Acids Co-Extraction by a Green Process
Source: Molecules. 2018 Apr 11;23(4):874. doi: 10.3390/molecules23040874 (PMC6017215; doi:10.3390/molecules23040874)
Supplement: Supplementary file 1 [file molecules-23-00874-s001.pdf]

Supplementary data

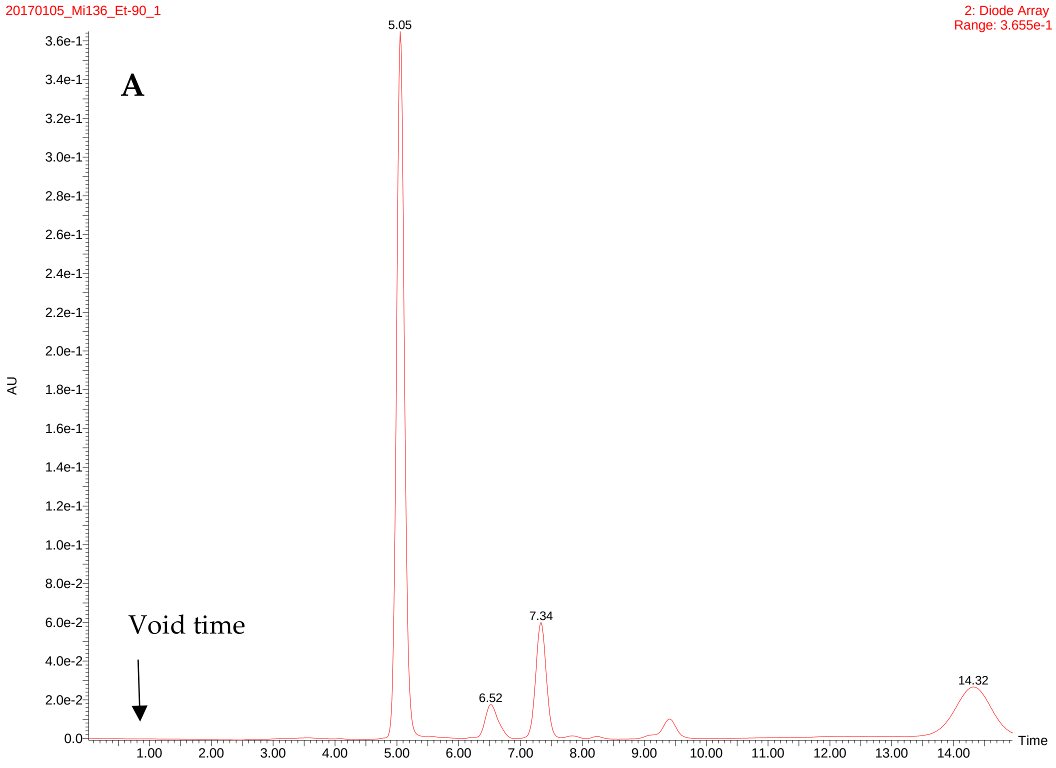

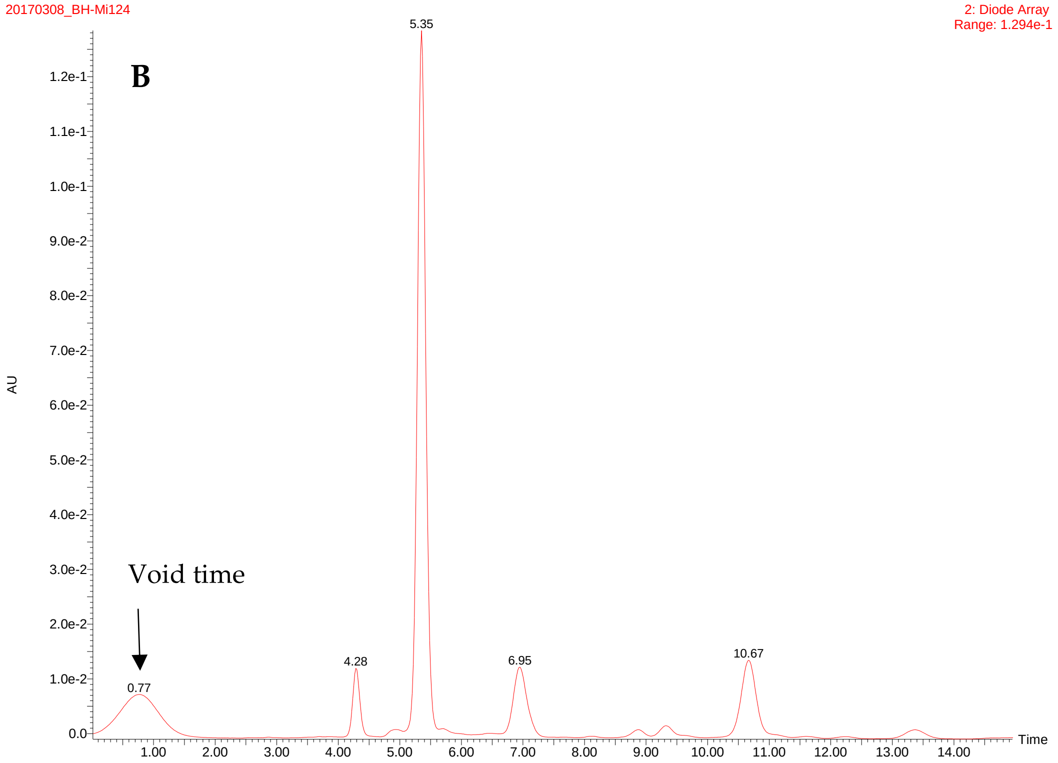

Figure S1. Chromatograms corresponding to ethanolic extracts of (A) *P. tricornutum* (RT fucoxanthin: 5.05 min) and (B) *T. lutea* (RT fucoxanthin: 5.35 min).

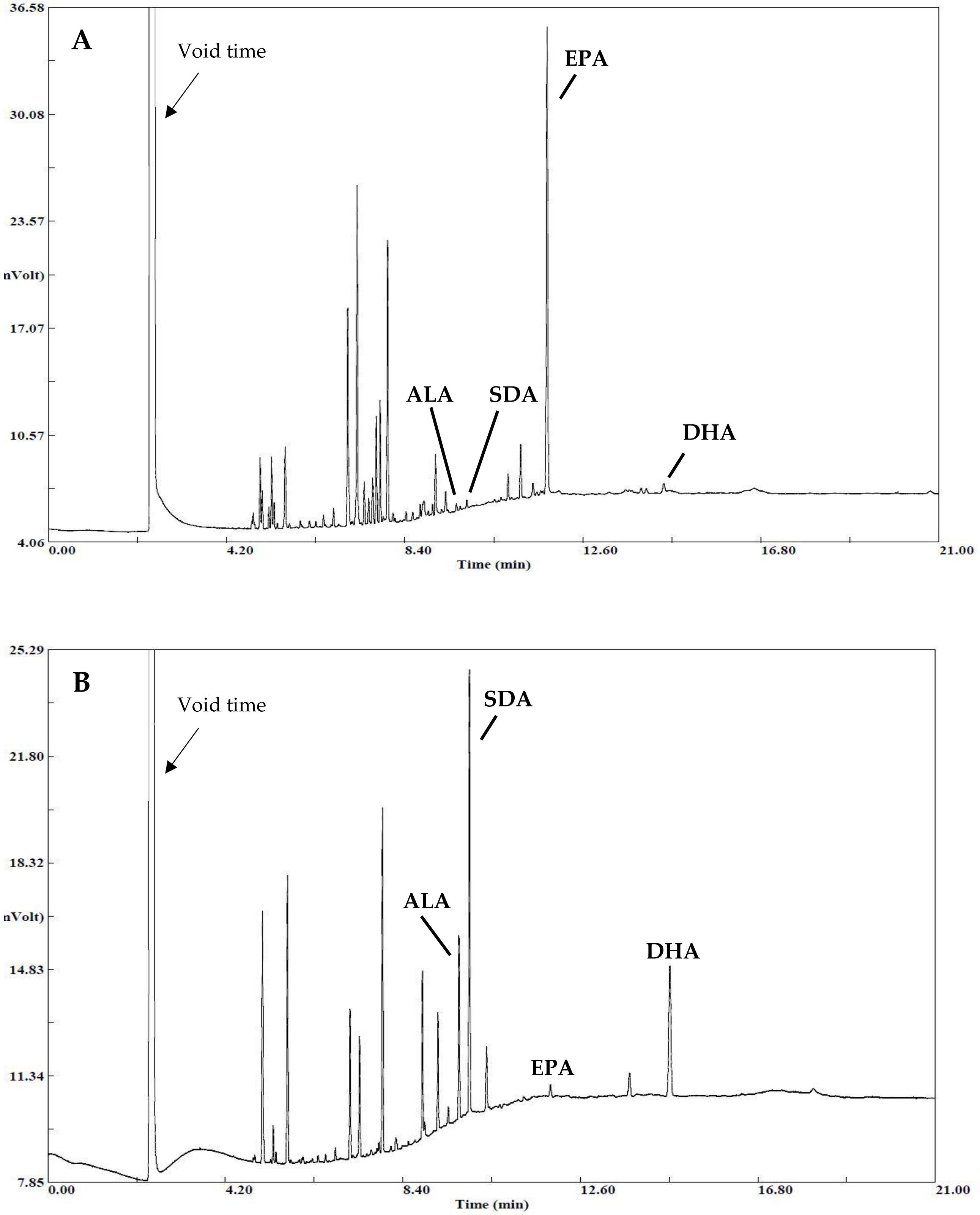

Figure S2. Chromatograms corresponding to direct transmethylation from biomass of (A) *P. tricornutum* (RT ALA: 9,62 min; SDA: 9,86 min; EPA: 11,75 min; DHA: 14,50 min) and (B) *T. lutea* (RT ALA: 9,73 min; SDA: 9,97 min; EPA: 11,90 min; DHA: 14,70 min).

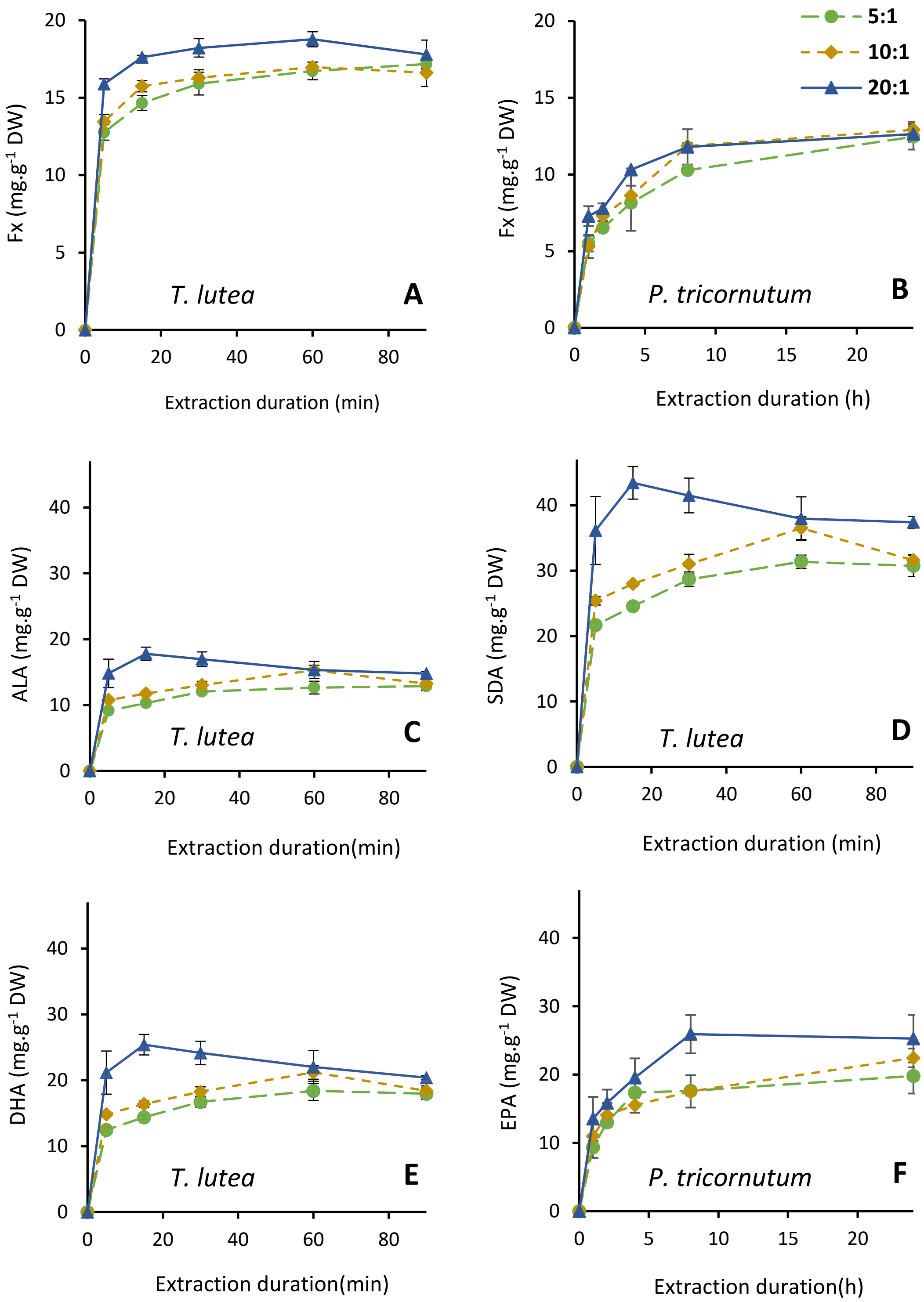

Figure S3. Influence of the solvent/biomass ratio (v/w) on the extraction kinetics and yield of fucoxanthin (A and B) and long chain polyunsaturated fatty acids (PUFAs), namely linolenic acid (ALA, C), Stearidonic acid (SDA, D), docosahexaenoic acid (DHA, E), eicosapentaenoic acid (EPA, F) from freeze-dried *Tisochrysis lutea* (A, C, D and E) and *Phaeodactylum tricornutum* (B and F) biomass. Extractions were carried out for 90 min at 30°C with 96% ethanol in a closed system with magnetic stirring (A, C, D and E) or for 24h at 40°C (B and F) (n = 3 replicates).
